# Rhinovirus Infection and Virus-Induced Asthma

**DOI:** 10.3390/v14122616

**Published:** 2022-11-24

**Authors:** Yuriko Hayashi, Mitsuru Sada, Tatsuya Shirai, Kaori Okayama, Ryusuke Kimura, Mayumi Kondo, Mitsuaki Okodo, Takeshi Tsugawa, Akihide Ryo, Hirokazu Kimura

**Affiliations:** 1 Department of Health Science, Gunma Paz University Graduate School of Health Sciences, Takasaki-shi 370-0006, Gunma, Japan; 2 Advanced Medical Science Research Center, Gunma Paz University Research Institute, Shibukawa-shi 377-0008, Gunma, Japan; 3 Department of Bacteriology, Gunma University Graduate School of Medicine, Maebashi-shi 371-8514, Gunma, Japan; 4 Department of Clinical Engineering, Faculty of Medical Technology, Gunma Paz University, Takasaki-shi 370-0006, Gunma, Japan; 5 Department of Medical Technology, Faculty of Health Sciences, Kyorin University, 5-4-1 Shimorenjaku, Mitaka-shi 181-8621, Tokyo, Japan; 6 Department of Pediatrics, School of Medicine, Sapporo Medical University, Sapporo-shi 060-8543, Hokkaido, Japan; 7 Department of Microbiology, School of Medicine, Yokohama City University, Yokohama-shi 236-0004, Kanagawa, Japan

**Keywords:** rhinovirus, virus-induced asthma, cytokines, airway tissue remodelling

## Abstract

While the aetiology of asthma is unclear, the onset and/or exacerbation of asthma may be associated with respiratory infections. Virus-induced asthma is also known as virus-associated/triggered asthma, and the reported main causative agent is rhinovirus (RV). Understanding the relationship between viral infections and asthma may overcome the gaps in deferential immunity between viral infections and allergies. Moreover, understanding the complicated cytokine networks involved in RV infection may be necessary. Therefore, the complexity of RV-induced asthma is not only owing to the response of airway and immune cells against viral infection, but also to allergic immune responses caused by the wide variety of cytokines produced by these cells. To better understand RV-induced asthma, it is necessary to elucidate the nature RV infections and the corresponding host defence mechanisms. In this review, we attempt to organise the complexity of RV-induced asthma to make it easily understandable for readers.

## 1. Introduction

Although the pathophysiology of asthma associated with viral infections is not fully understood, epidemiological findings have suggested that respiratory infections may be involved in the onset and/or exacerbation of asthma [1,2]. Asthma associated with viral infections is termed virus-induced asthma [3]. Several respiratory viruses can be involved, including human rhinovirus (RV), respiratory syncytial virus (RSV), and human parainfluenza virus; these viruses are causative agents in the onset and/or exacerbation of asthma. In particular, RV has been found to be associated with >60% of cases of asthma exacerbation in children [4].

The airway system consists of airway epithelial and connective tissues and smooth muscles [5]. These tissues contain blood vessels, microlymphoid tissues, and immune cells with various functions [5]. Therefore, the composition of the airway system is complex.

In all studies to date, the maintenance and homeostasis of the airway system have been proposed to be orchestrated by/associated with various immunological mediators, such as cytokines, from various cells in the airway system [6]. Virus-induced asthma has been suggested to disturb maintenance/homeostasis [7]. However, pleiotropy and the redundancy of cytokines are major obstacles in understanding virus-induced asthma [7]. In this review, to better understand the pathophysiology of RV-induced asthma, we describe the disease based on multifaceted findings related to RV virology and biological defence systems.

## 2. Pathophysiology of Asthma

The pathophysiology of asthma is characterised by airway inflammation and remodelling [8,9]. In asthmatics, airway inflammation is present persistently, not only during attacks [8]. Airway epithelial cells play a pivotal role in airway inflammation in asthma by acting as a barrier to the outside world and producing cytokines and chemokines [10]. Moreover, remodelling, i.e., simultaneous cell death and regeneration with smooth muscle thickening and fibrous tissue, results in hyperplasia. Thus, extensive remodelling may lead to airway stiffening and lumen narrowing, resulting in respiratory disorders [9].

## 3. Major Processes of Airway Tissue Remodelling in Virus-Induced Asthma

**Figure 1:**
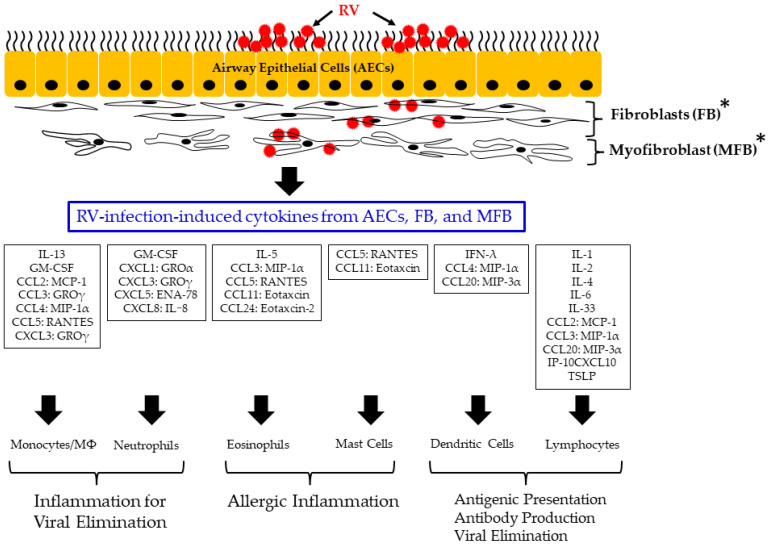
Schematic illustration of major processes of tissue remodeling in virus-induced asthma. * These data were only shown in an in vitro study.

As shown in Figure 1, the major processes of tissue remodelling in virus-induced asthma are as follows: (1) epithelial inflammatory responses, including cytokine and inflammatory factor production, against viral infection; (2) the activation of leukocytes (lymphocytes, neutrophils, eosinophils, mast cells, and monocytes/macrophages) and the production of cytokines/inflammatory factors from them; and (3) the proliferation of fibroblasts and smooth muscle cells triggered by the processes in (1) and (2). Therefore, to better understand airway remodelling induced by an RV infection, understanding RV’s virology and biological defence system, which involves various immunological responders such as chemokines/cytokines and cellular immunity against RV, is necessary [11].

## 4. RV Virology

RV is a non-enveloped virus that belongs to the family *Picornaviridae* and the genus *Enterovirus* and causes various respiratory symptoms and diseases, including rhinitis, sore throat, cough, bronchitis, and pneumonia [12]. RVs are classified into the following species: A (RV-A), B (RV-B), and C (RV-C) [13]. Previous genetic studies have further classified RV-A, RV-B, and RV-C into over 100, 30, and 50 genotypes, respectively [14]. The major viral antigen is viral protein 1 (VP 1), and the amino acid sequences of this protein show wide divergence, which accounts for the wide antigenicity of RVs and other enteroviruses, such as echoviruses [15,16]. The cellular receptors for RV-associated VP1 are intercellular adhesion molecule type-1 (ICAM-I: CD54), cadherin-related family member 3 (CDHR3), or low-density lipoprotein receptor (LDLR) [17]. These receptors are commonly expressed in airway epithelial cells [17]. Notably, a recent study suggested that RV-C induces not only the release of Th1 cytokines but also Th2 cytokines, including interleukin (IL)-4, -5, and -6, in virus-infected airway epithelial cells [18].

## 5. Obstacles to Understanding Cytokines in Virus-Induced Asthma

For a better understanding of asthma pathophysiology, cytokines have been classified into two major groups: Th1 and Th2. Th1 cytokines, such as IL-1β, tumour necrosis factor (TNF)-α, and interferon (IFN)-γ, are cytokines produced by Th1 lymphocytes. Similarly, cytokines that are produced by Th2 lymphocytes are called Th2 cytokines, which include IL-4, -5, and -13.

Most viral infections induce a dominant production of Th1 cytokines, such as IL-1β and IFNs. These cytokines may induce the production of antiviral antibodies, including IgM and IgG, by activating Th1 and B cells [19,20]. IL-12 and IFN-γ may induce naïve CD4^+^ T cells’ differentiation into Th1 cells and activate them [21]. Moreover, activated Th1 cells secrete IL-12 and IFN-γ, which activate macrophages and enhance their capacity for digestion and the presentation of antigens [22]. In contrast, allergens, such as food proteins from eggs and meat, may sometimes induce the production of Th2 cytokines [23,24]. These cytokines can activate allergic cells, such as eosinophils and mast cells, and induce IgE (reagin) production [25,26]. IgE binds to allergens, and this allergen–reagin complex activates mast cells and induces histamine release, thereby leading to anaphylaxis [27,28].

To better understand the pathophysiology and phenotypes of asthma, the Th1/Th2 balance paradigm has been widely used [29]. In this paradigm, eosinophilic asthma develops when Th2 cytokines and Th2 lymphocytes are predominant over Th1 cytokines and Th1 lymphocytes. In contrast, it is thought that Th1 predominance causes neutrophilic asthma and autoimmune diseases such as Crohn’s disease and rheumatoid arthritis [30]. However, this paradigm cannot fully explain virus-induced asthma because viral infection leads to Th1 dominance. Recently, it has been discovered that there are cytokines and lymphocytes that do not fit into this classical paradigm.

The function of group 2 innate lymphoid cells (ILC2s) in asthma has attracted much interest. ILC2s are innate immune cells with no antigen specificity that produce large quantities of Th2 cytokines [31]. ILC2s are activated by IL-33 that is released from injured airway epithelial cells and are associated with steroid resistance in asthmatics [31,32]. Thus, ILC2s play an essential role in eosinophilic asthma. Inflammation caused by ILC2s and Th2 lymphocytes is called type 2 inflammation and has been studied intensively [31].

Th17 cells have also attracted attention in recent years and are thought to be a new player in the pathophysiology of various inflammatory diseases [33]. Th17 lymphocytes produce IL-17, which can recruit neutrophils and, like Th1 lymphocytes, is involved in the onset and exacerbation of Crohn’s disease [30]. In the context of the pathophysiology of asthma, an in vivo study suggested that IL-17 may lead to steroid-resistant asthma [34]. Interestingly, Beale et al. reported that IL-25, a family member of IL-17, is necessary for rhinovirus-exacerbated allergic pulmonary inflammation [35].

In general, most cytokines show pleiotropy and redundancy [36]. Moreover, cytokines are aggregates with these characteristics and constitute a network. Therefore, understanding the relationship between cytokines and virus-induced asthma may be difficult. Typical representative examples of such cytokines are IFNs.

IFNs are classified into three groups in humans. Type I IFNs include IFN-α and -β, type II IFNs include IFN-γ, and type III IFNs include IFN-λ [37,38]. Type I IFNs can have an anti-viral effect by suppressing viral replication and activating natural killer cells to eliminate virus-infected cells. Type I IFNs also induce the release of various cytokines [39,40]. CCL5 release is induced by type I IFNs via the Janus kinase/signal transducer and activator of transcription (JAK/STAT) pathways and the activation of interferon-stimulated genes (ISGs) [41]. CCL5 is a crucial chemokine in the migration of CD8 T cells to the lungs and is associated with the typical IFN-γ-dominant Th1 response and anti-viral immunity [42,43]. On the other hand, CCL5 simultaneously induces strong chemotaxis in eosinophils [43,44,45]. Type III IFNs also have an anti-viral effect and regulate various cytokines [46]. When the lung tissue is infected with virus, type III IFNs are released and suppress viral replication without inducing inflammation [47]. Moreover, previous reports suggested that Th2 cytokine levels, including IL-4, IL-5, and IL-33, were decreased by type III IFNs [48,49]. Thus, IFNs activate not only Th1 inflammation but also type 2 inflammation.

In this review, we have tried to avoid this complication by explicating the relationships between the cytokines shown in Table 1 [50,51,52,53,54,55,56,57,58,59,60,61,62,63,64,65,66,67,68,69,70,71,72,73,74,75,76] and their biological activities. Moreover, we simply classify cytokines into Th1, Th2, and others [77,78].

## 6. Relationships between RV-Infection-Induced Cytokine Responses in Airway Epithelial Cells, Fibroblasts, and Myofibroblasts

The injured airway epithelial cells produce and release various cytokines, chemokines, and damage-associated molecular patterns (DAMPs) that can activate innate immune systems. For example, polyinosinic-polycytidylic acid [poly (I:C)], which is structurally similar to double-stranded RNA and is used instead of an RNA virus infection in vitro and in vivo, can induce the release of IFN-γ, TNF-α, IL-8, and CCL5 in bronchial epithelial cells [79,80,81]. Nakamoto et al. reported that flagellin, derived from *Pseudomonas aeruginosa*, induces the release of IL-6 and IL-8 in airway epithelial cells [82]. Thus, airway epithelial cells are not only barriers to environmental stimulations but also act as triggers for inflammation and the release of various signalling molecules. Moreover, the cytokines, chemokines, and DAMPs released from airway epithelial cells may induce the release of other cytokines or chemokines from fibroblasts, myofibroblasts, and leukocytes. For example, transforming growth factor β (TGF-β) derived from epithelial cells is important in the development of airway remodelling in refractory asthma and it induces IL-6 production in fibroblasts [83,84]. In this manner, the injury of airway epithelial cells by environmental factors can lead to cascades of cytokine and chemokine production.

Viral infection is one of the major causes of injury to airway epithelial cells. Airway epithelial cells perceive viral infection and virus-induced injury and activate immune responses. One of these responses is the activation of the innate immune system through pattern-recognition receptors (PRRs) [85]. Toll-like receptors (TLRs) are one of the most representative PRRs. TLR3 recognises viral double-stranded RNA, and previous reports have shown that cytokines and chemokines are produced in bronchial epithelial cells via TLR3 activation during viral infection [86,87]. TLR7/8 recognises single-stranded RNA. Several reports have suggested that various cytokines are produced in bronchial epithelial cells via TLR7/8 activation at the time of viral infection [88,89]. Other RNA-sensing PRRs include retinoic acid-inducible gene-I (RIG-I), MDA5 (melanoma differentiation-associated gene 5), and protein kinase R (PKR) [90]. The recognition of viral RNA by these molecules can also induce the release of various cytokines. A response other than cytokine production that can occur as a result of viral infection is the production of reactive oxygen species (ROS) in airway epithelial cells. ROS can induce apoptosis in airway cells [91]. Comstock et al. reported that viral infection and poly (I:C) stimulation promote ROS production in bronchial epithelial cells [92]. Thus, viral infection leads to various immune responses and promotes cascades of cytokine and chemokine production.

Among the viruses that can damage airway epithelial tissue, RV is a major pathogen, and is one that exacerbates asthma, as described above. As shown in Figure 1, RV can infect airway epithelial cells, leading to a cascade of cytokine and chemokine production [93,94,95]. Indeed, RV-infected epithelial cells can produce various T1 cytokines, including granulocyte colony-stimulating factor, IFN-γ, and TNF-α, and type2 cytokines including IL-4, IL-5, and IL-13 [96,97,98]. These cytokines may further induce the production of various cytokines in fibroblasts and myofibroblasts, irrespective of RV infection [99,100]. Moreover, fibroblasts and myofibroblasts infected with RV may also induce the release of cytokines, although this has been confirmed only in an in vitro study [94,98].

As mentioned above, RV infection may induce the production of various cytokines in airway component cells including epithelial cells, fibroblasts, and myofibroblasts [101]. These cytokines also activate leukocytes. Among them, MCP-1 (CCL2), MIP-1α (CCL3), and IP-10 (CXCL10) may activate lymphocytes; MCP-1 (CCL2), MIP-1α (CCL3), and RANTES (CCL5) may activate monocytes/macrophages; GROα (CXCL1), GROγ (CXCL3), ENA-78 (CXCL5), and IL-8 (CXCL8) may activate neutrophils; MIP-1α (CCL3), RANTES (CCL5), eotaxin (CCL11), and eotaxin-2 (CCL24) may activate eosinophils; RANTES (CCL5) and eotaxin (CCL11) may activate mast cells; and MIP-3α (CCL20) activates dendritic cells (DCs) [69,102,103]. Moreover, various chemokines act as chemoattractant proteins that induce the migration and recruitment of neutrophils, eosinophils, and monocytes/macrophages from adjacent vascular tissue to the RV infection foci [104,105].

## 7. Effector Functions of Leukocytes

Leukocytes are also associated with the pathophysiology of asthma. The effector functions of various leukocytes, such as neutrophils, monocytes/macrophages, eosinophils, and mast cells, are involved in degranulation, the generation of ROS, and phagocytosis [106]. These effector functions are triggered and enhanced by Th1 and/or Th2 cytokines and/or stimulation by various substances, such as platelet-activating factor derived from RV-infected cells [96]. Leukocytes interact with each other through such effector functions and the release of cytokines. Thus, leukocytes form a network among themselves.

DCs are activated by cytokines released from airway epithelial cells, take up allergens, and migrate to local lymph nodes. DCs present antigens via major histocompatibility gene complex class II molecules, and CD40-mediated stimulation can induce CD4 T cells to differentiate into Th1, Th2, or Th17 cells [107].

Th1 lymphocytes produce Th1 cytokines such as IFN-γ and TNF-α and activate monocytes/macrophages and lymphocytes (cytotoxic T cells) in order to kill virus-infected cells [88,89]. Alveolar macrophages play specific roles in the phagocytosis of various particles such as dust, bacteria, and viruses and have strong effector functions including ROS generation and the degranulation of myeloperoxidase [108]. Therefore, in the acute phase of airway infection by RVs, these effector functions of macrophages may be partly responsible for exacerbating virus-induced asthma [109,110]. In addition, Th1 cytokines activate neutrophils. In an in vivo study, activated neutrophils increased the expression of the high-affinity IgE receptor (FcεRI) on lung-conventional DCs [111]. Therefore, Th1 lymphocytes may contribute to accelerating type 2 inflammation.

Moreover, Th2 lymphocytes produce IL-4, IL-5, IL-13, and IL-33 and activate eosinophils and mast cells, leading to allergic reactions, including virus-induced asthma [112,113]. Mast cells release histamine and matrix metalloproteinases (MMPs), and they cause increased vascular permeability and bronchial smooth muscle contractions. Eosinophils contain toxic granules (i.e., major basic protein (MBP), eosinophil cationic protein (ECP), eosinophil-derived neurotoxin (EDN), and eosinophil peroxidase) and efficiently produce ROS upon various stimulations, such as that effected by cytokines, and enhanced effector functions by eosinophils can cause tissue injury and airway remodelling in RV-induced asthma [114,115].

Since the leukocyte network is complicated, the relationship between individual leukocytes is not fully understood. Therefore, it may be necessary to investigate the mechanism by which Th1 lymphocytes accelerate type 2 inflammation, which is unconsidered by the classical Th1/Th2 balance paradigm.

## 8. Differences between Asthmatics and Non-Asthmatics in Viral Infection

The production of cytokines and chemokines in the airway compartment and by various immune cells may occur in both asthmatics and non-asthmatics. It remains unclear what causes asthmatic airway inflammation. However, several theories have been considered in this respect.

First, a type 2 dominant environment in the airway accelerates type 2 inflammation in airway components. In airway epithelial cells, poly (I:C) stimulation induces greater CCL5 production with IL-4 and IL-13 pre-treatments than without [116]. In addition, sputum IL-13 levels in patients with poorly controlled asthma are higher than those in patients with well-controlled asthma. Thus, a Th2-predominant environment creates type 2 inflammation and may form a spiral that leads to asthma exacerbation in asthmatic patients.

Second, there are low levels of IFN-β in the bronchial epithelial cells of asthmatics [116]. Since IFNs play an essential role in viral elimination, asthmatics with low levels of IFN-β fail to eliminate viruses and develop worse asthma exacerbation. From this perspective, a clinical trial using nebulised IFN-β therapy was conducted and reported some efficacy [117].

Third, there are persistent viral infections in asthmatics. As described above, bronchial epithelial cells and airway macrophages in asthmatics have low levels of IFNs and fail to mount an effective innate immune response in the airway. Thus, viral clearance may be incomplete in the airways of asthmatics. Indeed, Wos et al. reported that RV was detected in the lower airway tissue of asthmatics more often than in non-asthmatics [118].

These points are controversial. The first point is very useful for understanding the pathophysiology of eosinophilic asthma. On the other hand, a multifaceted approach is important to understand the pathophysiology of asthma because it has variously different phenotypes. Thus, the differences between asthma and non-asthma phenotypes need to be more closely scrutinised.

## 9. Perspective

Although inhaled corticosteroid therapy improves the progression of asthma, it is still a major disease burden globally [119]. An RV infection is a common cause of asthma exacerbation. Thus, understanding the pathophysiology of virus-induced asthma, especially RV-related asthma, is essential for developing new treatment agents. As described in this review, the relationships between viral infection and the production of cytokines in the airway compartment have been clarified to some extent, such as those concerning IFN production and the cytokines induced by IFNs. However, the differences between a normal airway and an asthmatic airway are still unclear. Therefore, further studies may be needed to elucidate the differences between asthmatics and non-asthmatics with respect to viral infection.

## Figures and Tables

**Table 1:** Relationships among various cytokines, origins, and functions.

	Cytokines	Origin	Function	Ref. No.
Th1	IFN-γ	Activated T cell	Macrophage activation	[55,74]
	IFN-λ	Activated T cell	Dendritic cell activation	[75]
	TNF-α	Activated T cell, Monocyte/Macrophage	Promotes inflammatory cytokines	[50,76]
	IL-2	Activated T cell	T cell/NK cell activation	[51]
	GM-CSF	Activated T cell Monocyte/Macrophage, Fibroblast	Neutrophil/Macrophage activation	[52]
Th2	IL-4	Activated T cell	B cell/T cell activation IgE isotype switch	[53,58]
	IL-5	Activated T cell Eosinophil, Mast cell	Eosinophil proliferation/differentiation	[54,55]
	IL-6	Activated T cell	Induce B cells into antibody-producing cells T-cell differentiation	[56]
	IL-13	Activated T cell Mast cell	Macrophage activation IgE isotype switch	[57,58]
Others	IL-1	Monocyte/MΦ	T cell activation	[59]
	IL-33	Activated T cell Endothelial/Epithelial cell	Promoting Th2-associated cytokines	[60,61]
	CXCL1 GROα	Epithelial cells, Macrophage, Neutrophil	Neutrophil migration	[62]
	CXCL3 GROγ MIP-2β	Monocyte, Fibroblast	Neutrophil migration/adhesion	[63]
	CXCL5 ENA-78	Eosinophil	Neutrophil migration/activation	[64]
	CXCL8 IL-8	Monocyte/Macrophage, Fibroblast	Neutrophil migration	[62,65]
	CXCL10 IP-10	Monocyte, Fibroblast	T cell/NK cell activation	[66]
	CCL2 MCP-1	Monocyte/Macrophage, Fibroblast	Monocyte migration	[67]
	CCL3 MIP-1α	Monocyte/Macrophage, Activated T cell	Monocyte migration/infiltration Eosinophil/mast cell/Dendritic cell migration	[68]
	CCL4 MIP-1β	Monocyte/Macrophage, Activated T cell	Monocyte/T cell migration	[68]
	CCL5 RANTES	Monocyte/Macrophage, Fibroblast	Monocyte/lymphocyte/Eosinophil/mast cell migration	[69,70]
	CCL11 Eotaxin	Airway Epithelial Cells, Fibroblast	Eosinophil migration/degranulation Mast cell activation/migration	[71]
	CCL20 MIP-3α	Neutrophil, Natural Killer Cell	Dendritic cell activation/migration T cell/B cell migration	[72]
	CCL24 Eotaxin-2	Monocyte/Macrophage	Eosinophil migration	[71]
	TSLP	Epithelial cells	Promoting Th2-associated cytokines B cell activation	[73]

## Data Availability

Not applicable.
